# Long-term bresults of radiotherapy combined with nedaplatin and 5-fluorouracil for postoperative loco-regional recurrent esophageal cancer: update on a phase II study

**DOI:** 10.1186/1471-2407-12-542

**Published:** 2012-11-22

**Authors:** Keiichi Jingu, Haruo Matsushita, Ken Takeda, Rei Umezawa, Chiaki Takahashi, Toshiyuki Sugawara, Masaki Kubozono, Keiko Abe, Takaya Tanabe, Yuko Shirata, Takaya Yamamoto, Youjirou Ishikawa, Kenji Nemoto

**Affiliations:** 1 Department of Radiation Oncology, Tohoku University School of Medicine, 1-1 Seiryo-chou, Aoba-ku, Sendai, 980-8574, Japan; 2 Department of Radiation Oncology, Yamagata University School of Medicine, Yamagata, Japan

**Keywords:** Postoperative recurrent esophageal cancer, Chemoradiotherapy, Long-term results, Phase II study

## Abstract

**Background:**

In 2006, we reported the effectiveness of chemoradiotherapy for postoperative recurrent esophageal cancer with a median observation period of 18 months. The purpose of the present study was to update the results of radiotherapy combined with nedaplatin and 5-fluorouracil (5-FU) for postoperative loco-regional recurrent esophageal cancer.

**Methods:**

Between 2000 and 2004, we performed a phase II study on treatment of postoperative loco-regional recurrent esophageal cancer with radiotherapy (60 Gy/30 fractions/6 weeks) combined with chemotherapy consisting of two cycles of nedaplatin (70 mg/m^2^/2 h) and 5-FU (500 mg/m^2^/24 h for 5 days).

The primary endpoint was overall survival rate, and the secondary endpoints were progression-free survival rate, irradiated-field control rate and chronic toxicity.

**Results:**

A total of 30 patients were enrolled in this study. The regimen was completed in 76.7% of the patients. The median observation period for survivors was 72.0 months. The 5-year overall survival rate was 27.0% with a median survival period of 21.0 months. The 5-year progression-free survival rate and irradiated-field control rate were 25.1% and 71.5%, respectively. Grade 3 or higher late toxicity was observed in only one patient. Two long-term survivors had gastric tube cancer more than 5 years after chemoradiotherapy.

Pretreatment performance status, pattern of recurrence (worse for patients with anastomotic recurrence) and number of recurrent lesions (worse for patients with multiple recurrent lesions) were statistically significant prognostic factors for overall survival.

**Conclusions:**

Radiotherapy combined with nedaplatin and 5-FU is a safe and effective salvage treatment for postoperative loco-regional recurrent esophageal cancer. However, the prognosis of patients with multiple regional recurrence or anastomotic recurrence is very poor.

## Background

Extended radical esophagectomy with three-field (neck, mediastinum, and abdomen) lymph node dissection has been performed since the mid-1980’s, and it seems to have improved survival of patients with esophageal cancer [1-3]. However, there is recurrence in 27-52% of operated patients and loco-regional recurrence in 41.5-55% of patients with postoperative recurrence [3-9]. In 2006, we reported the effectiveness of radiotherapy and concurrent chemotherapy for postoperative recurrent esophageal cancer with a median observation period of 18.0 months [10]. Although the results were better than those of other studies using radiotherapy with or without chemotherapy, the observation period was not sufficient. Furthermore, there have been no prospective studies with a long-term observation period for patients with postoperative loco-regional recurrent esophageal cancer.

The purpose of the present study was to update the results of the phase II study of definitive radiotherapy with nedaplatin (CDGP) and 5-fluorouracil (5-FU) for loco-regional recurrent esophageal cancer after curative resection.

## Methods

The present study was performed between 2000 and 2004 in Tohoku University Hospital and two affiliated hospitals according to the following protocol.

All patients had histologically proven squamous cell carcinoma of the esophagus. Patient selection criteria included 1) 30 to 80 years of age, 2) Eastern Cooperative Oncology Group (ECOG) performance status of 0 to 3, 3) no other active cancer, 4) no serious cardiac, liver, or pulmonary disease, 5) creatinine clearance of more than 50 ml/min, 6) adequate bone marrow function (leukocyte count of 4000/μl, platelet count of 100,000/μl, 7) loco-regional recurrence (including para-aortic lymph node metastasis) without distant metastasis after no residual tumor (R0) resection by extended radical esophagectomy with three-field (neck, mediastinum, and abdomen) lymph node dissection, and 8) no previous therapy other than R0 resection.

Recurrence was diagnosed comprehensively by upper gastrointestinal endoscopy, ultrasonography, computed tomography (CT), physical findings and/or cytology.

### Radiotherapy

A linear accelerator (4 MV or 10 MV) was used as the X-ray source. The target volume was localized for radiotherapy in all patients by CT planning. The daily fractional dose of radiotherapy was 2.0 Gy, administered 5 days a week, and the total dose was 60.0 Gy. For 11 patients, a T-shaped field (including the bilateral supraclavicular, mediastinal and abdominal regions) was used. For the remaining 19 patients, extended local fields with a margin of 1 to 2 cm from the macroscopic tumor were used. After 40 Gy, the field was changed in all patients to avoid the spinal cord, and only macroscopic lesions were irradiated with a margin of 1 to 1.5 cm.

### Chemotherapy

**Figure 1:**
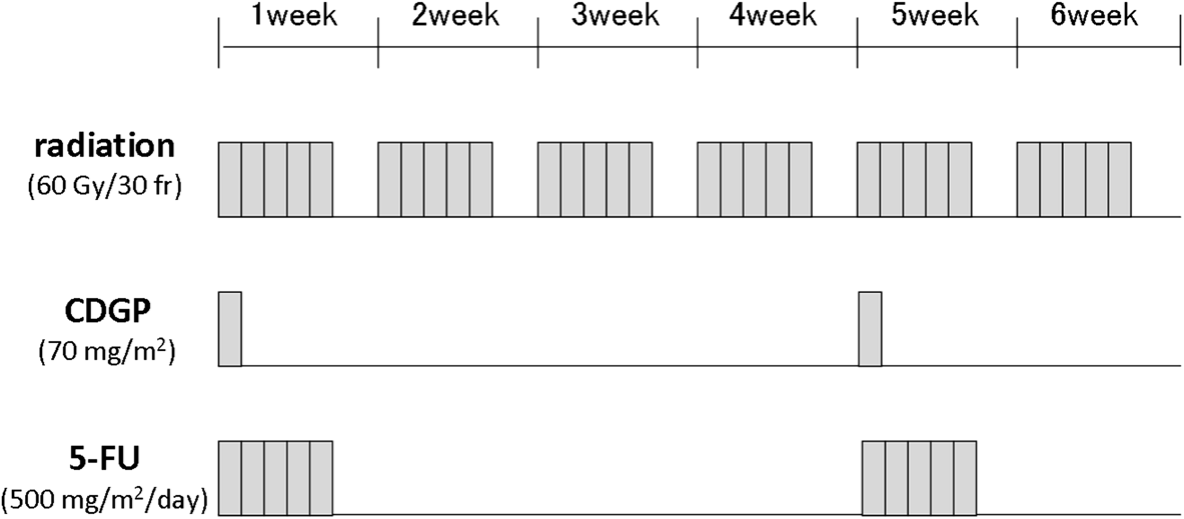
Schedule of the protocol of chemoradiotherapy.

Each cycle of chemotherapy consisted of 120-minute infusion of CDGP at 70 mg/m^2^ and a 5-day period of 5-FU at 500 mg/m^2^/day. This cycle of chemotherapy was repeated with an interval of 4 weeks, for a total radiotherapy dose of 60 Gy (Figure 1). When toxicity of grade 3 or higher was noted and prolonged, we suspended or discontinued chemotherapy or reduced the dose of CDGP alone or the dose of both CDGP and 5-FU by 25-30% in the subsequent cycle.

Completion of the regimen in this study was defined as completion of two cycles of full-dose CDGP + 5-FU for a total radiotherapy dose of 60 Gy without suspension of treatment.

### Endpoint

The primary endpoint of the present study was overall survival rate, and the secondary endpoints were progression-free survival rate, irradiated-field control rate and late toxicity.

### Follow-up

Follow-up evaluations were performed every 3–6 months for the first 2 years and every 12 months thereafter by endoscopy and CT.

Only progression disease (PD) according to Response Evaluation Criteria In Solid Tumors (RECIST) was defined as failure of the present regimen (relapse again).

### Toxicity

Toxicity was graded according to the Common Terminology Criteria for Adverse Events (CTCAE v3.0). An adverse effect at more than 90 days after completion of chemoradiotherapy was defined as late toxicity.

### Statistics

Survival estimates were calculated using the Kaplan-Meier method from the first date of radiotherapy, and differences were evaluated by the log-rank test. Age (65 years or less vs. more than 65 years), preoperative stage (I-II vs. III-IV: Union for International Cancer Control 1997 (UICC1997)), time interval between surgery and recurrence (12 months or less vs. more than 12 months), pre-radiotherapy performance status (0–1 vs. 2–3), radiation field (local alone vs. T-shaped), acute tumor response according to RECIST (complete regression (CR) ~ partial regression (PR) vs. stable disease (SD) ~ PD), number of cycles of chemotherapy (one vs. two), pattern of recurrence (anastomotic vs. non-anastomotic) and number of recurrent regions (one region vs. multiple regions) were entered into the log-rank test. A p value of less than 0.05 was considered significant. All analyses were performed using SPSS 20.0.

The present study protocol was reviewed and approved by our institutional review board (No. 2012-1-129), and informed consent was obtained from each patient before conducting the treatment.

## Results

From 2000 to 2004, a total of 30 patients (29 males, 1 female; median age, 64 years; age range, 50 to 72 years) were enrolled in this phase II study. Patient characteristics are shown in Table 1. The rate of completion of this regimen without reduction of chemotherapy was 76.7%. The median observation period was 72 months (range, 16.5 to 125.5 months) for patients who survived. At the last observation date, 18 of the 30 patients had relapse again. Twenty-two of the 30 patients died: deaths were due to progression disease in 19 patients, intercurrent diseases in 2 patients and an iatrogenic cause in one patient.

**Table 1:** **Patients**’ **characteristics**

**patient number**	**age**	**gender**	preoperative stage (UICC*1997)	**time interval between surgery and recurrence (months)**	**PS (ECOG†)**	**recurrent regions**	**field**	**number of cycles of chemo**	tumor response (RECIST*)	**irradiated field recurrence (yes/no)**	**recurrence (yes/no)**	**dead/alive**	**survival period (months)**
1	70	male	IIA	6	1	subclavicular/mediastinal	T-shaped	2	PR	yes	yes	dead	6.5
2	64	male	III	6	1	mediastinal	T-shaped	2	SD	yes	yes	dead	14
3	64	male	IIA	47	1	anastomosis/subclavicular/mediastinal	T-shaped	2	SD	no	yes	dead	5
4	50	male	III	12	1	abdominal	local	2	PR	no	yes	dead	8
5	64	male	III	7	1	mediastinal/abdominal	local	1	SD	no	yes	dead	5
6	62	male	III	13	2	anastomosis	T-shaped	2	PR	no	yes	dead	4
7	55	male	III	5	3	anastomosis/subclavicular/abdominal	local	2	PR	no	yes	dead	5.5
8	61	female	IIB	28	0	mediastinal	T-shaped	2	C R	no	no	alive	54
9	64	male	III	8	0	anastomosis/mediastinal	T-shaped	2	C R	yes	yes	dead	23.5
10	62	male	IIB	22	1	abdominal	local	2	PR	no	yes	dead	10
11	65	male	I	102	0	mediastinal	T-shaped	2	C R	no	no	alive	90
12	60	male	IV B	6	1	subclavicular	local	2	PR	no	no	alive	98
13	72	male	III	7	0	abdominal	local	2	PR	no	yes	dead	39
14	67	male	III	46	0	subclavicular	T-shaped	2	C R	no	no	alive	49.5
15	54	male	n.a.	4	0	abdominal	local	2	SD	no	yes	dead	12
16	68	male	III	19	1	anastomosis/subclavicular	local	2	PR	no	yes	dead	33
17	69	male	III	6	1	anastomosis/subclavicular	local	2	PR	no	yes	dead	28.5
18	68	male	n.a.	36	2	mediastinal	local	2	PR	no	no	dead	18.5
19	62	male	III	10	1	mediastinal	local	2	PR	no	no	alive	16.5
20	66	male	IV B	66	0	subclavicular	local	1	PR	yes	yes	dead	116
21	57	male	III	11	3	anastomosis	T-shaped	2	PR	no	no	dead	8
22	52	male	III	23	1	mediastinal	T-shaped	1	PD	no	yes	dead	6
23	56	male	I	54	1	anastomosis	local	1	PR	no	no	dead	6
24	62	male	IIB	23	0	mediastinal	local	2	PR	no	no	alive	125.5
25	71	male	I	4	1	subclavicular	local	2	SD	no	no	alive	107
26	61	male	I	83	1	abdominal	local	2	SD	yes	yes	dead	42
27	72	male	III	16	0	mediastinal	local	1	SD	no	no	alive	29
28	71	male	III	6	2	anastomosis	T-shaped	2	C R	yes	yes	dead	13.5
29	63	male	III	39	0	mediastinal	local	2	PR	no	yes	dead	45
30	72	male	III	4	1	mediastinal	local	2	PR	no	yes	dead	21

* UICC: Union for International Cancer Control.

† ECOG: Eastern Cooperative Oncology Group.

# RECIST: Response Evaluation Criteria in Solid Tumors.

**Figure 2:**
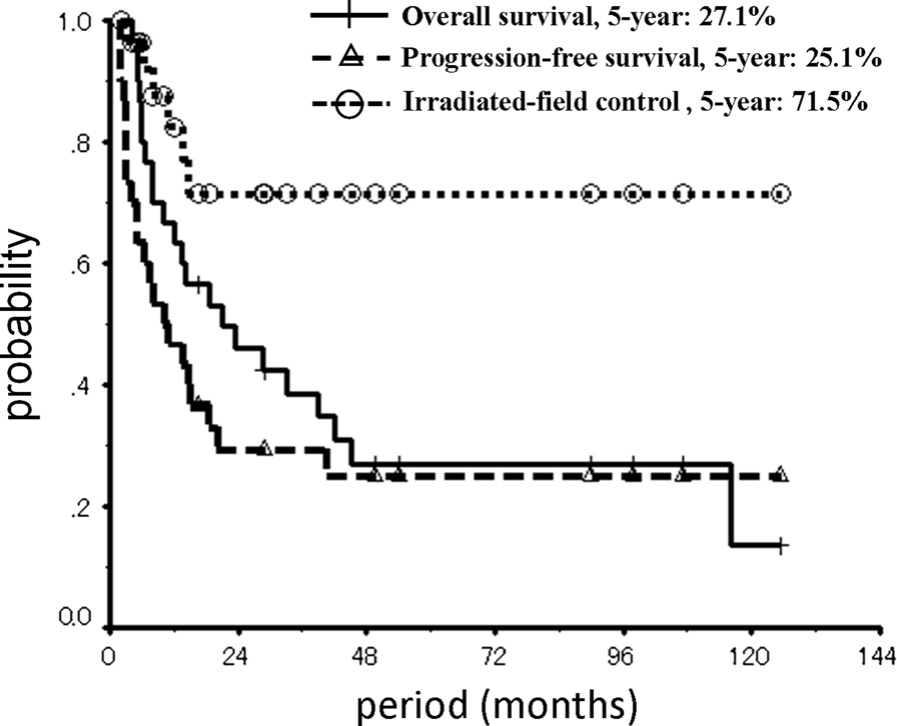
Overall survival, progression-free survival and irradiated-field control rates in patients with postoperative loco-regional recurrent esophageal cancer (Kaplan-Meier method).

The 3-year and 5-year overall survival rates were 38.4% (95% confidence interval (CI) = 20.8-56.5) and 27.0% (95% CI = 10.3-43.7), respectively, with a median survival period of 21.0 months (95% CI = 2.5-39.5). The 3-year and 5-year progression-free survival rates were 29.3% (95% CI = 12.8-45.9) and 25.1% (95% CI = 9.1-41.2), respectively, and both of the 3-year and 5-year irradiated-field control rates were 71.5% (95% CI = 51.8-91.2) (Figure 2).

Acute toxicities have already been reported in our previous report [10]. As the major late toxicity, only one patient had grade 3 or higher toxicity. The patient died 6 months after completion of the protocol due to serious pericardial effusion. There was no other grade 3 or higher late toxicity, although grade 1 or 2 focal pulmonary fibrous change, pericardial effusion and/or pleural effusion were often observed. Although not toxicity, there were 2 patients who developed gastric tube cancer, which could be controlled with endoscopic submucosal dissection.

In log-rank test, the difference between overall survival rate in performance status (p = 0.007), pattern of recurrence (p = 0.014) and number of recurrent lesions (p = 0.003) were statistically significant (Table 2).

**Table 2:** Prognostic factors for overall survival

**factor**	**group**	**No.**	**median survival period (month)**	**log-rank p value**
performance status	0-1	25	33.0	0.007
	2-3	5	8.0	
age	≥65	12	33.0	0.172
	<65	18	10.0	
preoperative stage (UICC§ 1997)	I-II	9	42.0	0.229
	III- IV	21	21.0	
number of cycles of chemotherapy	1	5	6.0	0.577
	2	25	23.5	
time interval between surgery and recurrence	≤12	15	14.0	0.176
	>12	15	42.0	
tumor response (RECIST*)	CR-PR	22	23.5	0.466
	SD-PD	8	12.0	
field	local	19	33.0	0.480
	T-shaped	11	13.5	
number of recurrent regions	one	23	39.0	0.014
	multiple	7	6.5	
pattern of recurrence	anastomotic	9	8.0	0.003
	non-anastomotic	21	42.0	

§UICC: Union for International Cancer Control, *RECIST: Response Evaluation Criteria in Solid Tumors.

## Discussion

There have been some studies on the effectiveness of radiotherapy with or without chemotherapy for treatment of postoperative recurrent esophageal cancer. In those studies, even the 2-year survival rates were only 15-31% with short-term observation (Table 3) [11-15]. We previously reported preliminary results of the present study, which were excellent. Here, updated results with long-term observation are reported. Although the results are worse than those in the past preliminary report [10], the results of the current regimen remain one of the best achievements for patients with postoperative loco-regional recurrent esophageal cancer.

**Table 3:** **Contents and results of radiotherapy** (**with or without chemotherapy**) **for postoperative recurrent esophageal cancer in past studies**

**author**	**year**	**No**.	**regimen**	**median observation period**	**2-year survival rate**	**5-year survival rate**
JL Raoul^12)^	1995	24	RT† + CDDP*+5-FU	14 months	17.1%	n.a. ‡
K Nemoto^13)^	2001	33	RT alone (21) or RT + CDDP+ 5-FU (12)	n.a.	15%	n.a.
Y Nishimura^11)^	2003	13	RT + CDDP+5-FU	9.5 months	19%	n.a.
Y Shioyama^14)^	2007	82	RT ± chemotherapy	n.a.	22%	11%
K Maruyama^15)^	2011	23	RT ± chemotherapy	n.a.	31%	24%
Current study	2012	30	RT + CDGP§+ 5-FU	72 months	46.0%	27.0%

†RT: radiotherapy, * CDDP: cisplatin, § CDGP: nedaplatin, # 5-FU: 5-fluorouracil, ‡n.a.: not available.

Recurrence after surgery can now be detected earlier due to improvements in the resolution of CT. It might improve recently the treatment results for postoperative loco-regional recurrent esophageal cancer. FDG-PET/CT, which enables detection of recurrence at an earlier stage than that by only CT, has been used frequently since the mid-2000’s for esophageal cancer, and the prognosis of loco-regional recurrent esophageal cancer may therefore be further improved.

We previously reported excellent results of chemoradiotherapy for solitary lymph node metastasis after curative surgery for esophageal cancer [16], and the results for patients with recurrence in one region were also significantly better than those for patients with recurrence in more than one region in the present study. This supports our hypothesis that the concept of oligo-recurrence [17] might also be applicable to postoperative esophageal cancer, especially in cases without anastomotic recurrence.

The appropriate irradiation field for postoperative loco-regional recurrent esophageal cancer has not been clarified. In the present study, there were some patients in whom recurrent lesions could not be controlled, but there were no patients who had regional lymph node recurrence after chemoradiotherapy. In our previous study on solitary lymph node metastasis [16], there were 2 patients who showed other lymph node metastases after chemoradiotherapy, but both of those patients had undergone irradiation with a T-shaped field. Furthermore, patients who were treated with a T-shaped field had a significantly higher rate of adverse events than did patients who were treated with a local field [10]. In the present study, there was no late toxicity in the gastric tube even with 60 Gy; however, there have been some reports of problems in the gastric tube caused by obstruction of blood flow [18,19], which can be induced by radiation. Therefore, we do not recommend irradiation with a prophylactic field such as a T-shaped field for postoperative loco-regional recurrent esophageal cancer. Furthermore, Zhang et al. reported that results for patients treated with 60 Gy or more were significantly better than results for patients treated with less than 60 Gy in patients with postoperative loco-regional recurrent esophageal cancer [20], and we reported in 2001 that one of our patients died of necrosis of the gastric tube 6 months after the end of 66 Gy radiotherapy [13]. According to those reports and the present results, the appropriate radiation dose for loco-regional recurrent esophageal cancer might be 60 Gy.

In the present study, 2 of the 5 patients who survived for more than 5 years after chemoradiotherapy had gastric tube cancer, which fortunately could be completely resected by endoscopic submucosal resection (ESD). In the past, when esophageal cancer patients seldom survived for a long time, the occurrence of gastric tube cancer was considered to be infrequent [21]. Recent improvements in the survival of patients after esophagectomy, however, have led to increasing occurrence of gastric tube cancer [22,23]. Bamba et al. reported that the 10-year cumulative incidence of gastric tube cancer after esophagectomy was 8.6% [24], and they described the possible cause of the high incidence of gastric cancer after esophagectomy. Asian people have a high rate of Helicobacter pylori infection and therefore have a high risk of gastric cancer [25]. Upper gastrointestinal endoscopy once or twice per year is recommended for follow-up after treatment for postoperative loco-regional recurrent esophageal cancer. Gastric tube cancer is one of the major complicating diseases in patients who survive for a long time after treatment of esophageal cancer.

## Conclusions

The present protocol of radiotherapy combined with CDGP and 5-FU is a safe and effective salvage treatment for postoperative loco-regional recurrent esophageal cancer. However, the prognosis of patients with multiple regional recurrence or anastomotic recurrence is very poor.

## Competing interests

The authors declare that they have no competing interests.

## Author’s contributions

KJ drafted the manuscript and performed statistical analysis. KN participated in the study design and coordination. HM, CT, KT, TS, RU, MK, KA, TT, TY, YS and YI performed the chemoradiotherapy and the follow-up. All of the authors have read and approved the final manuscript.

## Pre-publication history

The pre-publication history for this paper can be accessed here:

http://www.biomedcentral.com/1471-2407/12/542/prepub
